# On the Determinants and Outcomes of Passion for Playing Pokémon Go

**DOI:** 10.3389/fpsyg.2018.00316

**Published:** 2018-03-15

**Authors:** Gábor Orosz, Ágnes Zsila, Robert J. Vallerand, Beáta Böthe

**Affiliations:** ^1^Institute of Psychology, Eötvös Loránd University (ELTE), Budapest, Hungary; ^2^Institute of Cognitive Neuroscience and Psychology, Research Centre for Natural Sciences, Hungarian Academy of Sciences, Budapest, Hungary; ^3^Doctoral School of Psychology, Eötvös Loránd University (ELTE), Budapest, Hungary; ^4^Laboratoire de Recherche sur le Comportement Social, Université du Québec à Montréal, Montreal, QC, Canada

**Keywords:** gaming motives, harmonious passion, obsessive passion, Pokémon Go, impulsivity, structural equation modeling

## Abstract

In 2016, Pokémon Go became the most popular smartphone game. Despite the increasing popularity of this augmented reality game, to date, no studies have investigated passion for playing Pokémon Go. On the theoretical basis of the Dualistic Model of Passion (DMP), our goal was to investigate the associations between Pokémon Go playing motives, passion, and impulsivity. A total of 621 Pokémon Go players participated in the study (54.9% female; *M*_age_ = 22.6 years, *SD*_age_ = 4.4). It was found that impulsivity was more strongly associated with obsessive passion (OP) than with harmonious passion (HP). HP was associated with adaptive motives (i.e., outdoor activity, social, recreation, and nostalgia), while OP was associated with less adaptive motives (i.e., fantasy, escape, boredom, competition, and coping). Therefore, in line with the DMP, HP and OP for playing Pokémon Go can predict an almost perfectly distinguished set of adaptive or maladaptive playing motives, and OP has a noteworthy relationship with impulsivity as a determinant.

## Introduction

### The Pokémon Go phenomenon: history and playing motives

Pokémon Go has become an increasingly popular augmented reality game, particularly among youth (Dorward et al., 2017; Kamel Boulos et al., 2017). After the first release of Pokémon Go in July 2016, 21 million active players engaged in the world of Pokémon within 1 week, and this game has become the most popular smartphone application, beating the most frequently visited social networking sites such as Facebook, Twitter, or Instagram (Dorward et al., 2017).

Pokémon Go is a massively multiplayer online role-playing game (MMORPG) in which players can find and capture virtual Pokémon species in their real environment (Kamel Boulos et al., 2017). The captured species are added to the player's “Pokédex,” a catalog of caught Pokémon. Captured Pokémon can be trained and evolved into stronger forms, and players can challenge others to gym battles (Dorward et al., 2017). The world of “Pocket Monsters” was first introduced to Game Boy players in the mid-1990s. The original story was adapted into an anime series later which attracted millions of young viewers in the 2000s (Katsuno and Maret, 2004). The Pokémon Go application is the latest media product of the Pokémon franchise (Dorward et al., 2017).

Due to the massive success of Pokémon Go, there has been a considerable research interest into the positive and negative sites of usage in terms of physical and mental health (e.g., Althoff et al., 2016; Ayers et al., 2016; Tateno et al., 2016). While a number of studies found associations between Pokémon Go playing activity and several physical and mental health benefits such as reducing sedentary behavior (Nigg et al., 2017), promoting health behaviors (e.g., exercising or walking) (Kaczmarek et al., 2017), and decreasing social withdrawal and anxiety relating to social interactions (Tateno et al., 2016; Kogan et al., 2017), other studies pointed out to a few concerns related to the inappropriate use of the game such as distracting drivers (Ayers et al., 2016) or being lost in unexplored areas (Dorward et al., 2017). Furthermore, it was found that Pokémon Go players reported higher psychological distress than those workers who had not played Pokémon Go (Watanabe et al., 2017).

In sum, previous research points out to relevant behavioral changes in Pokémon Go players as they incorporated playing into their daily routines and spent considerable amount of time outside, discovering remote areas as well as getting a deeper knowledge of their natural environment (Dorward et al., 2017). Contrary to prior expectations about the quick decay in the popularity of this game, there are still millions of active players worldwide of this game and updates (e.g., Pokémon Go Plus, released in January 2017). In light of the important number of Pokémon players worldwide, better understanding of the associations between the use patterns of this augmented reality game and players' psychological characteristics is still needed.

Prior studies investigated the motives for playing online games (e.g., Yee, 2006; Fuster et al., 2014), whereas Demetrovics et al. (2011) offered an integrated model comprising seven dimensions: social (i.e., playing with others), escapism (i.e., escaping reality), competition (i.e., defeating others), coping (i.e., coping with real-life problems), skill development (i.e., improving skills), fantasy (i.e., immersing in another world), and recreation (i.e., relaxing) motives. Zsila et al. (2017) extended this model with three Pokémon Go-specific motives: outdoor activity (i.e., playing outside), nostalgia (i.e., reliving old memories), and boredom (i.e., passing time playing). Another study by Yang and Liu (2017) identified seven Pokémon Go playing motives: fun, friendship maintenance, relationship initiation, exercise, achievement, escapism, and nostalgia.

Among these motives, we can identify adaptive and maladaptive ones. For instance, escaping reality and competition were identified as a strong predictor of problematic online gaming (Király et al., 2015). Furthermore, achievement and social motives were found to be related to psychological well-being (Fuster et al., 2014). However, the adaptive vs. maladaptive role of nostalgia is less evident. According to Routledge et al. (2013), nostalgia is related to elevated psychological health and well-being and it promotes adaptive psychological functioning. However, in a recent, gaming-related study, it was weakly and positively associated with loneliness (Yang and Liu, 2017). Increased physical activity associated with Pokémon Go playing was also found to be related to psychological and physical well-being (Althoff et al., 2016; Howe et al., 2016; Kamboj and Krishna, 2017).

Orosz et al. (2016) suggested that the quality of engagement in different screen-based activities—such as playing Pokémon Go—can be measured and distinguished by the two forms of passion. The adaptive and maladaptive nature of the engagement or motives toward online and screen-based activities can largely depend on the respective type of passion for the given activity.

### The dualistic model of passion

According to Vallerand (2010, 2015), passion refers to the engagement in a self-defining activity that one loves, finds important, and invests considerable amount of time and energy in it. The Dualistic Model of Passion (DMP) distinguishes between harmonious passion (HP) and obsessive passion (OP). HP is an adaptive form of engagement in an activity, since the person is able to maintain coherence between his/her preferred activity and other life activities. In contrast, OP is associated with the lack of control over a particular activity, leading to a rigid involvement and conflict between the self and other daily life activities (Vallerand et al., 2003; Vallerand, 2010). The present study focuses on the relationship between Pokémon Go playing motives and impulsivity within the empirically well-established DMP theoretical framework (for review see Vallerand, 2010, 2015; Curran et al., 2015).

In gaming literature, Wang and Chu (2007) found that OP was related to problematic gaming, whereas HP was unrelated to it. It was also found that HP for massively multiplayer online games predicted adaptive outcomes such as positive affect and vitality, while OP was associated with low level of need satisfaction, negative affect, excessive gaming, over-engagement, and physical and psychological addiction-like symptoms (Lafreniere et al., 2009; Przybylski et al., 2009; Stoeber et al., 2011; Orosz et al., 2016). Thus, based on previous findings in the field of online gaming, we expect that HP would be related to adaptive motives for playing augmented reality games such as Pokémon Go, whereas OP would be related to maladaptive motives.

### Impulsivity

The personality-related determinants of passion have not yet been investigated extensively in prior research. The relevant studies mainly focused on social determinants (e.g., Mageau et al., 2009; Bonneville-Roussy et al., 2011; Fernet et al., 2014). Only a few studies investigated personality traits as possible determinants of passion (e.g., Vallerand et al., 2006; Tosun and Lajunen, 2009; Orosz et al., 2016). Orosz et al. (2016) found that impulsivity can be one relevant personality trait underlying passion as it was positively related to OP but unrelated to HP.

According to the model of impulsivity by Whiteside and Lynam (2001), impulsivity comprises urgency (i.e., the tendency of engaging in impulse behaviors under negative emotional conditions in order to alleviate these negative feelings and without taking into consideration the potentially harmful long-term effects and consequences); lack of perseverance (i.e., inability to remain focused on a difficult or boring task); lack of premeditation (i.e., difficulty in considering the consequences of an act), and sensation seeking (i.e., tendency to pursue new, exciting activities). Later Billieux et al. (2012) complemented this model by distinguishing negative and positive forms of urgency. Based on this, we propose that people who can hardly resist temptations are more likely to engage in OP for playing Pokémon Go. They might play Pokémon Go in order to quickly and easily alleviate their negative feelings. People with lower levels of monotony tolerance might also develop OP for playing Pokémon Go when they are bored, and the reward system of the game drives them to engage in this activity repetitively.

### The aim of the present study

Despite the increasing popularity of Pokémon Go, relatively little research attention has been paid to the psychological background of playing this augmented reality game (Kaczmarek et al., 2017; Zsila et al., 2017). Considering that millions of active users worldwide still play Pokémon Go in 2017 (statista.com, 2017), the psychological background and motives associated with harmonious and OP for playing this game can be a relevant topic of investigation. Therefore, accumulating knowledge about psychological processes behind playing the most popular augmented reality game (in terms of impulsivity, motives, and passion) can be extremely beneficial if we move beyond gaming and think about work and private life-related aspects of this augmented reality game (Brohm et al., 2017). The aim of the present study was to explore the association of Pokémon Go playing motives with the two passion types (i.e., HP and OP). In the present study we aimed to put emphasis on the detailed outcomes of passion for playing Pokémon Go. We expected that HP would be positively related to adaptive Pokémon Go playing motives—social, skill development, and outdoor activity motives. Conversely, OP was expected to be positively related to escapism, competition and boredom motives. The exploration of these associations in the theoretical framework of passion would contribute to a more nuanced distinction of adaptive and maladaptive playing motives. Furthermore, we also investigated the relationship between passion types and one potential determinant of passion, namely impulsivity. Based on the findings of Orosz et al. (2016) with screen-based activities, we hypothesized that impulsivity would be positively related to OP but unrelated to HP.

## Materials and methods

### Participants and procedure

Ethical approval was gained from the Institutional Review Board of the Eötvös Loránd University, and the study was performed in accordance with the Declaration of Helsinki. The research was conducted using an online questionnaire. First, participants were informed about the aims and the content of the study. Second, they were assured that they could stop the participation without any consequences whenever the filling process was uncomfortable or unpleasant for them. The data collection occurred in July 2016. Participants were recruited from the largest Hungarian anime (*n* = 4) and gamer (*n* = 3) communities on Facebook (comprising about 2,000–8,000 members).

A total of 621 Hungarian Pokémon Go players participated in this study (54.90% female), aged between 18 and 54 years (*M*_age_ = 22.57 years, *SD*_age_ = 4.37). Participants spent 10.42 h per week on average playing Pokémon Go during the week preceding the data collection. Nearly half of them, 48.79% played Pokémon Go daily, whereas 38.65% played 2–6 times per week, and 12.55% played weekly or rarely. The vast majority of players (97.75%) played Pokémon Go on their mobile phone, whereas 2.25% played on their tablet. In the present study we used the same sample as in the Zsila et al.'s (2017) article. In the previous paper the factor structure of the MOGQ-PG was examined. However, in the present paper we intended to examine a specific relationship pattern regarding impulsivity, passion, and playing motives.

### Measures

#### Sociodemographic and Pokémon go-related information

Data regarding major demographics were collected including age and gender. Furthermore, data were obtained on the time spent playing Pokémon Go, frequency of playing, and the preferred platform (e.g., mobile phone).

#### Motives for online gaming questionnaire-Pokémon Go extension (MOGQ-PG)

The motives of Pokémon Go players were assessed using the MOGQ-PG (Zsila et al., 2017). The MOGQ-PG appeared to be a good choice as (1) it is based on qualitative studies, (2) it had strong theoretical and scientific background regarding its MOGQ part, (3) it had Pokémon Go-specific factors (including Outdoor Activity, Nostalgia, and Boredom), (4) it had appropriate within network validity (good model fit indices despite it includes 10 factors [Sample 1: CFI = 0.963; TLI = 0.958; RMSEA = 0.057]; [Sample 2: CFI = 0.965; TLI = 0.960; RMSEA = 0.054]), (5) it had appropriate internal consistency (all factors had higher Cronbach's alpha than 0.7), (6) besides it can comprehensively assess very diverse motivational factors, it is relatively short.

The 37-item scale comprises 10 subscales: Social (four items, “Because I can meet many different people,” α = 0.89), Escape (four items, “Because gaming helps me to forget about daily hassles,” α = 0.85), Competition (four items, “Because I enjoy competing with others,” α = 0.92), Coping (four items, “Because it helps me get rid of stress,” α = 0.80), Skill Development (four items, “Because it improves my skills,” α = 0.87), Fantasy (four items, “Because I can be in another world,” α = 0.86), Recreation (three items, “Because it is entertaining,” α = 0 77), Outdoor Activity (four items, “Because it provides the daily dose of exercise,” α = 0.92), Nostalgia (three items, “Because it reminds me of my childhood,” α = 0.92), and Boredom (three items, otherwise I would be bored, α = 0.78). Each item on the MOGQ-PG is rated on a 5-point Likert-scale (1 = almost never/never, 2 = some of the time, 3 = half of the time, 4 = most of the time, 5 = almost always/always).

#### The passion scale

The Hungarian version of the Passion Scale (Tóth-Király et al., 2017), developed by Vallerand et al. (2003) and Marsh et al. (2013), comprises six items on HP (“Playing Pokémon Go is in harmony with the other activities in my life,” α = 0.82), and six items on OP (“I have almost an obsessive feeling for playing Pokémon Go,” α = 0.88). In this study, the items of the Passion Scale focused on Pokémon Go. Participants indicated their level of agreement with the statements on a 7-point Likert scale (1 = do not agree at all, 7 = very strongly agree).

#### The short UPPS-P impulsive behavior scale (SUPPS-P)

The SUPPS-P Impulsive Behavior Scale (Billieux et al., 2012; Zsila et al., 2017) comprises 20 items that assess impulsivity on five dimensions: Negative Urgency (four items, e.g., “When I am upset I often act without thinking,” α = 0.80); Positive Urgency (four items, e.g., “When I am really excited, I tend not to think on the consequences of my actions.” α = 0.75); Lack of Perseverance (four items, e.g., “I finish what I start.,” α = 0.71); Lack of Premeditation (four items, e.g., “I usually think carefully before doing anything,” α = 0.81); Sensation Seeking (four items, e.g., “I generally seek new and exciting experiences and activities,” α = 0.75). The items were translated into Hungarian following the protocol of Beaton et al. (2000). Participants rated each item on a 4-point Likert scale (1 = Agree Strongly, 4 = Disagree Strongly). The total score of impulsivity in the structural regression model was computed by averaging the scores of the five subscales.

### Statistical analysis

Data analyses were performed with IBM SPSS for Windows, version 20.0 (IBM SPSS Inc., Chicago, Illinois) and Mplus 7.3 (Muthén and Muthén, 1998-2015) using a weighted least squares estimator (WLSMV) considering the non-normal distribution of a number of variables. Structural regression analysis within structural equation modeling (SEM) was used to investigate the associations between impulsivity, HP, OP, and Pokémon Go playing motives. The following fit indices were used to estimate the goodness of fit of the model to the data (Bentler, 1990; Brown, 2015): the Comparative Fit Index (CFI; ≥ 0.95 good, ≥ 0.90 acceptable), the Tucker–Lewis index (TLI; ≥ 0.95 good, ≥ 0.90 acceptable), and the Root-Mean-Square Error of Approximation (RMSEA; ≤ 0.06 good, ≤ 0.08 acceptable) with its 90% confidence interval.

Drawing on previous studies (e.g., Carbonneau et al., 2008; Orosz et al., 2016), parcels were used as indicators for the Passion Scale. Parcels are aggregated items that can be applied in models comprising a high number of latent and manifest variables. An important prerequisite of parceling is the unidimensionality of the scales (e.g., Bandalos and Finney, 2001; Matsunaga, 2008). Following the factorial algorithm of Rogers and Schmitt (2004), exploratory factor analysis was used, and three parcels were created for each of the two passion factor. For HP, parcel 1 consisted of items 5 and 6; parcel 2 consisted of items 3 and 8; and parcel 3 consisted of items 1 and 10. For OP, parcel 1 consisted of items 7 and 11; parcel 2 consisted of items 4 and 12; and parcel 3 consisted of items 2 and 9.

## Results

Descriptive statistics and inter-factor correlations are presented in Table 1.

**Table 1:** Descriptive statistics and inter-factor correlations of the subscales of included assessment instruments.

**Scales**	**Range**	***M***	***SD***	**1**	**2**	**3**	**4**	**5**	**6**	**7**	**8**	**9**	**10**	**11**	**12**	**13**	**14**	**15**	**16**	**17**
1. UPPS negative urgency	1–4	2.38	0.77	–																
2. UPPS positive urgency	1–4	2.51	0.70	−0.65^***^	–															
3. UPPS lack of perseverance	1–4	1.85	0.57	0.18^***^	−0.17^***^	–														
4. UPPS lack of premeditation	1–4	1.71	0.60	0.31^***^	−0.32^***^	0.57^***^	–													
5. UPPS sensation seeking	1–4	2.34	0.68	−0.17^***^	0.33^***^	0.12^**^	0.03	–												
6. UPPS impulsivity	1–4	2.16	0.30	0.33^***^	0.09^*^	0.69^***^	0.64^***^	0.58^***^	–											
7. Harmonious passion	1–7	3.70	1.31	0.00	−0.06	0.04	0.06	−0.09^*^	−0.03	–										
8. Obsessive passion	1–7	1.82	1.02	0.12^**^	−0.20^***^	0.20^***^	0.16^***^	0.10^*^	0.07	0.50^***^	–									
9. MOGQ-PG coping	1–5	2.27	0.96	0.14^***^	−0.16^***^	0.04	−0.01	−0.13^**^	−0.05	0.40^***^	0.43^***^	–								
10. MOGQ-PG nostalgia	1–5	3.57	1.32	0.02	−0.06	−0.03	−0.04	−0.16^***^	−0.11^**^	0.27^***^	0.20^***^	0.33^***^	–							
11. MOGQ-PG social	1–5	2.34	1.11	−0.04	−0.03	−0.05	−0.07	−0.20^***^	−0.17^***^	0.37^***^	0.30^***^	0.37^***^	0.26^***^	–						
12. MOGQ-PG competition	1–5	2.45	1.24	0.08	−0.16^***^	0.03	−0.01	−0.28^***^	−0.16^***^	0.27^***^	.32^***^	.36^***^	0.23^***^	0.43^***^	–					
13. MOGQ-PG skill development	1–5	1.95	0.95	0.10^*^	−0.16^***^	0.00	−0.03	−0.19^***^	−0.12^**^	0.42^***^	0.42^***^	0.61^***^	0.29^***^	0.39^***^	0.36^***^	–				
14. MOGQ-PG fantasy	1–5	2.23	1.16	0.16^***^	−0.23^***^	.11^**^	0.06	−0.13^**^	−0.02	0.35^***^	0.44^***^	0.58^***^	0.32^***^	0.27^***^	0.29^***^	0.54^***^	–			
15. MOGQ-PG recreation	1–5	4.00	0.87	−0.01	−0.05	−0.01	−0.02	−0.05	−0.06	0.52^***^	0.30^***^	0.52^***^	0.37^***^	0.37^***^	0.31^***^	0.40^***^	0.36^***^	–		
16. MOGQ-PG outdoor activity	1–5	3.00	1.24	0.02	−0.05	−0.03	−0.06	−0.06	−0.08^*^	0.46^***^	0.29^***^	0.44^***^	0.21^***^	0.41^***^	0.22^***^	0.48^***^	0.27^***^	0.50^***^	–	
17. MOGQ-PG escapism	1–5	2.02	1.06	0.15^***^	−0.17^***^	0.09^*^	0.03	−0.07	0.01	0.32^***^	0.43^***^	0.71^***^	0.22^***^	0.24^***^	0.23^***^	0.48^***^	.65^***^	0.39^***^	0.26^***^	–
18. MOGQ-PG boredom	1–5	2.80	1.12	0.15^***^	−0.20^***^	0.17^***^	0.07	−0.07	0.05	0.20^***^	0.25^***^	0.49^***^	0.22^***^	0.21^***^	0.26^***^	0.35^***^	0.37^***^	0.37^***^	0.26^***^	0.45^***^

*UPPS, UPPS Impulsivity Scale; MOGQ-PG, Motives for Online Gaming Questionnaire-Pokémon Go extension; M, mean; SD, standard deviation*.

* p < 0.05;

** p < 0.01;

*** *p < 0.001*.

**Figure 1:**
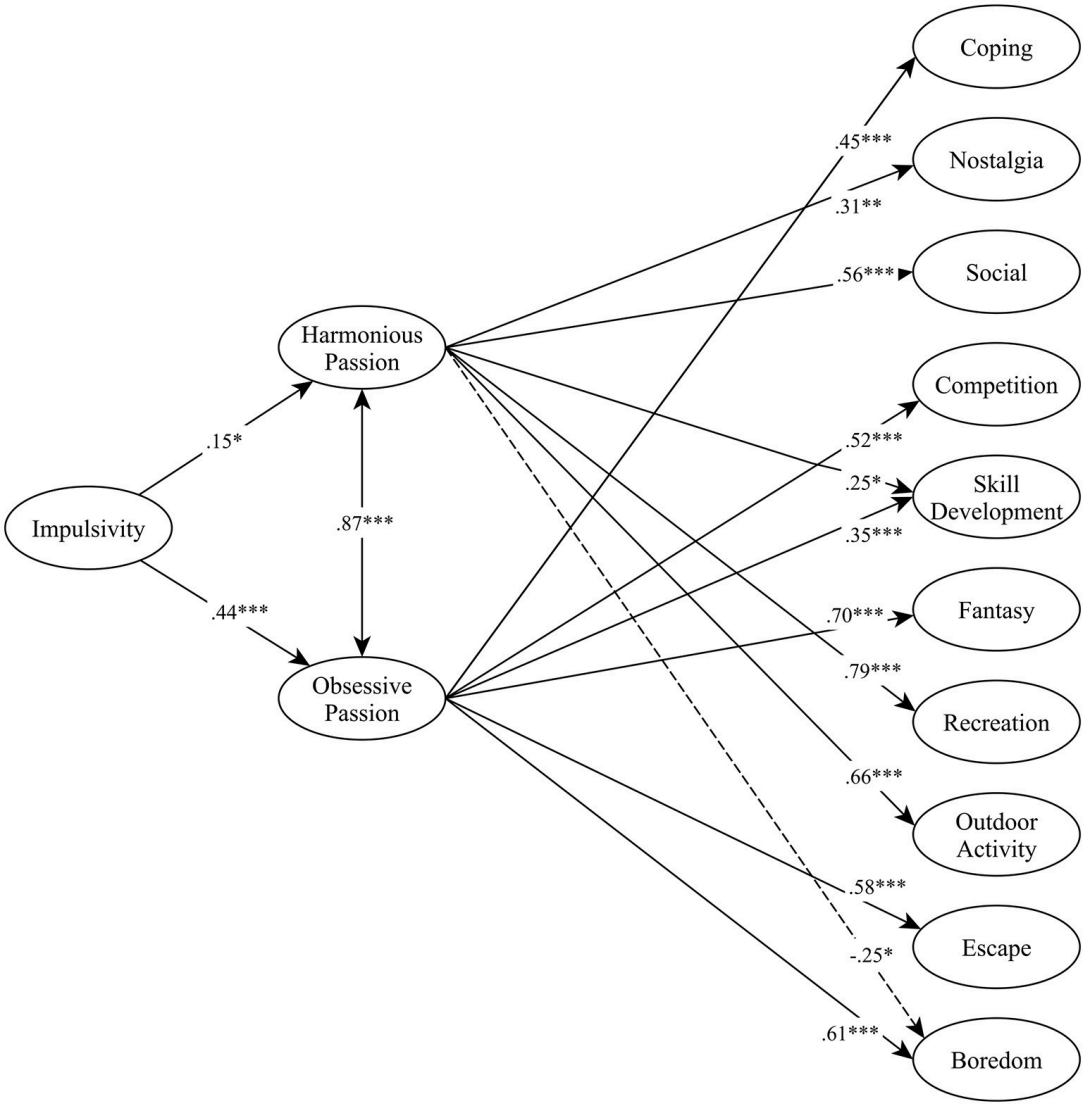
Harmonious and Obsessive passion as mediators between Impulsivity and Pokémon Go playing motives. Numbers indicate standardized betas; ^*^p < 0.05; ^**^p < 0.01; ^***^p < 0.001.

The results supported the hypothesized model [CFI = 0.951, TLI = 0.945, RMSEA = 0.049, (90% CI.047–0.052)], as seen in Figure 1. In the structural regression model, impulsivity was positively associated with both OP (β = 0.44, *p* < 0.001) and HP (β = 0.15, *p* = 0.002). As expected, the strength of this association was stronger for OP than for HP.

The findings almost perfectly supported the expected associations between the two types of passion and playing motives. More specifically, as expected, only HP was positively related to the Social (β = 0.56, *p* < 0.001), Nostalgia (β = 0.31, *p* = 0.009), Recreation (β = 0.79, *p* < 0.001), and Outdoor activity (β = 0.66, *p* < 0.001) motives. Conversely, as expected, OP was uniquely and positively related to Boredom (β = 0.61, *p* < 0.001), Coping (β = 0.45, *p* < 0.001), Competition (β = 0.52, *p* < 0.001), Fantasy (β = 0.70, negatively associated with Boredom (β = −0.25, *p* = 0.048). Only one Pokémon Go expected, HP was negatively associated with Boredom (β = −0.25, *p* = 0.048). Only one Pokémon Go playing motive was associated positively with both types of passion: Skill Development the presence of an almost perfect distinction between adaptive and maladaptive motives along HP and OP. Overall, these results reveal the presence of an almost perfect distinction between adaptive and maladaptive motives along HP and OP.

## Discussion

The goal of the present study was to investigate the relationship between passion, impulsivity and different motives concerning today's most popular augmented reality game, Pokémon Go. According to the results, impulsivity was more strongly associated with OP than with HP for playing Pokémon Go, which is partly in line with our hypothesis as we did not expect significant link between HP and impulsivity. In line with our expectations, OP was positively associated with several maladaptive motives such as escapism, coping, competition, boredom, and fantasy (as a less evidently maladaptive motive). Conversely, also in line with our expectations, HP was associated with several healthy or adaptive motives such as social, recreation, and outdoor activity motives. In sum, similar to other online or screen-related activities (Orosz et al., 2016), along with the DMP, the present model provided further support for the differentiated roles of HP and OP in terms of its personality determinants and motives.

HP was related to social and nostalgia motives, while these motives were not related to OP. These patterns were consistent with the association patterns reported by Fuster et al. (2014), who found that socialization, achievement, and exploration gaming motives were related to HP. However, nostalgia was related to HP, which contradicted the findings of a previous study by Yang and Liu (2017) on Pokémon Go playing motives who found a weak, positive link between loneliness and nostalgia. However, this result is in line with the more general notions of Routledge et al. (2013) concerning the adaptive function of nostalgia, and with those studies highlighting the importance of nostalgia in enhancing psychological well-being by fostering self-continuity and social connectedness (e.g., Routledge et al., 2013; Sedikides et al., 2016). The positive association between HP and these two motives drew the attention to the possible beneficial psychological consequences of playing Pokémon Go which may increase the sense of social connectedness by creating a social network that allows players to share and relive childhood memories of the world of Pokémon.

Passion can also facilitate engagement in behaviors that promote health-related activities such as physical activities (Vallerand, 2015). Positive consequences of HP were identified in prior studies such as self-development, physical and mental health (Lafreniere et al., 2009; Carpentier et al., 2012; Orosz et al., 2016). In the present case, health promotion lies in the nature of augmented reality games as they facilitate outdoor activities by blurring the line between real and virtual worlds, thus making the latter more interesting. On the basis of the results, we assume that if one has a HP for playing Pokémon Go, (s)he is motivated to play Pokémon Go for going outside and this physical activity can be beneficial for the player's health. Regarding mental health, this relationship pattern (HP➔Social) allows to initiate and maintain social relationships with different people (see also Kaczmarek et al., 2017). Therefore, on the basis of these results we might assume that HP for playing Pokémon Go may contribute to the players' physical and mental health. These results are in line with the Pikachu effect of Kaczmarek et al. (2017), who found that players with stronger health motives had more health benefits in terms of more time spent outside and increased physical activity. Harmonious passion for playing this AR game can be a mid-level construct behind this adaptive outcome.

According to prior studies, OP predicted negative health outcomes such as problematic or addiction-like symptoms (e.g., loss of control over the activity, Orosz et al., 2016), negative emotions (Przybylski et al., 2009), and health-risk behaviors (Vallerand et al., 2003). In light of prior studies and the present motivational correlates of OP, players with OP may be less motivated to play Pokémon Go to improve their mental or physical well-being. In the present study, OP was strongly related to escapism and boredom motives. According to previous results, playing online games in order to escape from real life problems can lead to problematic use (Király et al., 2015). Therefore, players who engage in Pokémon Go in order to escape from reality may be at risk of developing a problematic gaming behavior. Coping and competition were also expected to be related to OP, similar to the more general online gaming results of Király et al. (2015). The association of boredom with OP could be explained by the rigid, unsatisfying involvement in an activity, as was described by Vallerand et al. (2003) in their passion model. Furthermore, it was found that fantasy is positively related to OP. On the basis of the positive relationship pattern between OP, coping, escapism and fantasy, we may suppose that fantasy can also be interpreted as a creative internal form of escapism.

In line with previous findings (Orosz et al., 2016), impulsivity was positively associated with OP. However, impulsivity was also related to HP in the present study. According to prior studies, impulsivity can be interpreted as a risk factor for different problems in many fields, including health-risk behaviors (Vallerand et al., 2003), compulsive buying (Billieux et al., 2008), binge eating (Fischer and Smith, 2008; Peterson and Fischer, 2012), and Internet-related addictions (Mottram and Fleming, 2009). In the present study, both HP and OP were related to impulsivity, although this personality trait appeared to have a stronger relationship with OP than with HP. These results suggest that impulsivity may lead someone to rigidly engage in a behavior that is not necessarily problematic *per se*. However, playing Pokémon Go repeatedly at ill-advised times may lead to conflict with other aspects of one's life thereby to personal problems (e.g., neglecting one's studies) or interpersonal conflicts (e.g., neglecting one's romantic partner).

## Limitations

This study is not without its limitations. Due to the sampling method, players in this study may not be representative of the entire population of Pokémon Go players. Furthermore, since the assessment instruments in the present study were specific to Pokémon Go playing, information regarding individuals who do not play the game were not collected. Therefore, comparisons with a non-player group cannot be made. In addition, casual inferences cannot be established due to the cross-sectional nature of the study. Furthermore, the direction of associations could be reversed, thus alternative models should be tested in future studies. Another limitation is that Pokémon Go-specific motives can differ from the motives of other augmented reality games (e.g., nostalgia). Finally, the data collection was carried out at the time of Pokémon Go's peak popularity. Therefore, further research is needed not only on this particular augmented reality game but on the role of passion in popular games in general.

## Conclusion

The present study aimed to contribute to a deeper understanding of passion for the most popular augmented reality game, Pokémon Go. It was found that different playing motives are linked to different underlying passion constructs, which lead players to divergent experiences that may predict possible positive and negative consequences on the long run. Thus, the exploration of players' motives can lead to an advanced knowledge of Pokémon Go playing practices, which can unfold either healthy or maladaptive use of Pokémon Go. Therefore, the early identification of these motives related to maladaptive psychological mechanisms (e.g., OP) can indicate the need for intervention efforts to reduce the psychological harms of a problematic gaming behavior. The popularity of this game makes it reasonable to examine either positive or negative behavioral consequences of usage as millions of players engage daily in this augmented reality game in recent times.

Finally, the present study provided further evidence for the generalizability of the DMP by demonstrating the divergence of the two passion constructs with regard to playing motives in a relatively large sample of Pokémon Go players.

## Author contributions

GO and ÁZ substantially contributed to study design, data gathering, data analyses, interpretation of the results, and manuscript writing; BB substantially contributed to the data gathering, RV substantially contributed to the interpretation of the results, and revising the manuscript. All authors commented on the draft and contributed to the final version, approved the final version of the manuscript, and agreed to be accountable for all aspects of the work.

### Conflict of interest statement

The authors declare that the research was conducted in the absence of any commercial or financial relationships that could be construed as a potential conflict of interest.

## References

[B1] Althoff T. White R. W. Horvitz E. (2016). Influence of Pokémon Go on physical activity: study and implications. J. Med. Internet Res. 18:315. 10.2196/jmir.6759 27923778 PMC5174727

[B2] Ayers J. W. Leas E. C. Dredze M. Allem J. P. Grabowski J. G. Hill L. (2016).Pokémon Go – A new distraction for drivers and pedestrians. JAMA Intern. Med. 176, 1865–1866. 10.1001/jamainternmed.2016.6274 27635638

[B3] Bandalos D. L. Finney S. J. (2001). Item parceling issues in structural equation modeling, in New Developments and Techniques in Structural Equation Modeling, eds Marcoulides G. A. Schumacker R. E. (Mahwah, NJ: Lawrence Erlbaum Associates), 269–296.

[B4] Beaton D. E. Bombardier C. Guillemin F. Ferraz M. B. (2000). Guidelines for the process of cross-cultural adaptation of self-report measures. Spine 25, 3186–3191. 10.1097/00007632-200012150-00014 11124735

[B5] Bentler P. M. (1990). Comparative fit indexes in structural models. Psychol. Bull. 107, 238–246. 10.1037/0033-2909.107.2.238 2320703

[B6] Billieux J. Rochat L. Ceschi G. Carré A. Offerlin-Meyer I. Defeldre A. C.. (2012). Validation of a short French version of the UPPS-P impulsive behavior scale. Comp. Psychiatry 53, 609–615. 10.1016/j.comppsych.2011.09.001 22036009

[B7] Billieux J. Rochat L. Rebetez M. M. L. Van der Linden M. (2008). Are all facets of impulsivity related to self-reported compulsive buying behavior? Pers. Indiv. Differ. 44, 1432–1442. 10.1016/j.paid.2007.12.011

[B8] Bonneville-Roussy A. Lavigne G. L. Vallerand R. J. (2011). When passion leads to excellence: the case of musicians. Psychol. Music 39, 123–138. 10.1177/0305735609352441

[B9] Brohm D. Domurath N. Glanz-Chanos V. Grunert K. G. (2017). Future trends of augmented reality, in Augmented Reality for Food Marketers and Consumers, ed Loijens L. W. S. (Wageningen Academic Publishers), 97–104.

[B10] Brown T. A. (2015). Confirmatory Factor Analysis for Applied Research. New York, NY: Guilford Publications.

[B11] Carbonneau N. Vallerand R. J. Fernet C. Guay F. (2008). The role of passion for teaching in intrapersonal and interpersonal outcomes. J. Educ. Psychol. 100, 977–987. 10.1037/a0012545

[B12] Carpentier J. Mageau G. A. Vallerand R. J. (2012). Ruminations and flow: why do people with a more harmonious passion experience higher well-being? J. Happiness Stud. 13, 501–518. 10.1007/s10902-011-9276-4

[B13] Curran T. Hill A. P. Appleton P. R. Vallerand R. J. Standage M. (2015). The psychology of passion: A meta-analytical review of a decade of research on intrapersonal outcomes. Motiv. Emot. 39, 631–655. 10.1007/s11031-015-9503-0

[B14] Demetrovics Z. Urbán R. Nagygyörgy K. Farkas J. Zilahy D. Mervó B.. (2011). Why do you play? the development of the motives for online gaming questionnaire (MOGQ). Behav. Res. Methods 43, 814–825. 10.3758/s13428-011-0091-y 21487899

[B15] Dorward L. J. Mittermeier J. C. Sandbrook C. Spooner F. (2017). Pokémon Go: benefits, costs, and lessons for the conservation movement. Conserv. Lett. 10, 160–165. 10.1111/conl.12326

[B16] Fernet C. Lavigne G. L. Vallerand R. J. Austin S. (2014). Fired up with passion: investigating how job autonomy and passion predict burnout at career start in teachers. Work Stress 28, 270–288. 10.1080/02678373.2014.935524

[B17] Fischer S. Smith G. T. (2008). Binge eating, problem drinking, and pathological gambling: linking behavior to shared traits and social learning. Pers. Indiv. Differ. 44, 789–800. 10.1016/j.paid.2007.10.008

[B18] Fuster H. Chamarro A. Carbonell X. Vallerand R. J. (2014). Relationship between passion and motivation for gaming in players of massively multiplayer online role-playing games. Cyberpsychol. Behav. Soc. Netw. 17, 292–297. 10.1089/cyber.2013.0349 24611801

[B19] Howe K. B. Suharlim C. Ueda P. Howe D. Kawachi I. Rimm E. B. (2016). Gottacatch'em all! Pokémon GO and physical activity among young adults: difference in differences study. BMJ 355:i6270. 10.1136/bmj.i6270 27965211 PMC5154977

[B20] Kaczmarek L. D. Misiak M. Behnke M. Dziekan M. Guzik P. (2017). The Pikachu effect: social and health gaming motivations lead to greater benefits of Pokémon GO use. Comput. Hum. Behav. 75, 356–363. 10.1016/j.chb.2017.05.031

[B21] Kamboj A. K. Krishna S. G. (2017). Pokémon GO: an innovative smartphone gaming application with health benefits. Prim. Care Diabetes 11, 397–399. 10.1016/j.pcd.2017.03.008 28457897

[B22] Kamel Boulos M. N. Lu Z. Guerrero P. Jennett C. Steed A. (2017). From urban planning and emergency training to Pokémon Go: applications of virtual reality GIS (VRGIS) and augmented reality GIS (ARGIS) in personal, public and environmental health. Int. J. Health Geogr. 16:7. 10.1186/s12942-017-0081-0 28219378 PMC5319160

[B23] Katsuno H. Maret J. (2004). Localizing the Pokémon TV: series for the American market, in Pikachu's Global Adventure: The Rise and Fall of Pokémon, ed Tobin J. (Durham: Duke University Press), 80–107.

[B24] Király O. Urbán R. Griffiths M. D. Ágoston C. Nagygyörgy K. Demetrovics Z. (2015). The mediating effect of gaming motivation between psychiatric symptoms and problematic online gaming: an online survey. J. Med. Internet Res. 17:e88. 10.2196/jmir.3515 25855558 PMC4405620

[B25] Kogan L. Hellyer P. Duncan C. Schoenfeld-Tacher R. (2017). A pilot investigation of the physical and psychological benefits of playing Pokémon GO for dog owners. Comput. Hum. Behav. 76, 431–437. 10.1016/j.chb.2017.07.043

[B26] Lafrenière M. A. Vallerand R. J. Donahue E. G. Lavigne G. L. (2009). On the costs and benefits of gaming: the role of passion. Cyber Psychol. Behav. 12, 285–290. 10.1089/cpb.2008.0234 19366320

[B27] Mageau G. A. Vallerand R. J. Charest J. Salvy S.-J. Lacaille N. Bouffard T. (2009). On the development of harmonious and obsessive passion: the role of autonomy support, activity specialization, and identification with the activity. J. Pers. 77, 601–646. 10.1111/j.1467-6494.2009.00559.x 20078732

[B28] Marsh H. W. Vallerand R. J. Lafreniere M. A. K. Parker P. Morin A. J. S. Carbonneau N.. (2013). Passion: does one scale fit all? construct validity of two-factor passion scale and psychometric invariance over different activities and languages. Psychol. Assess. 25, 796–809. 10.1037/a0032573 23647035

[B29] Matsunaga M. (2008). Item parceling in structural equation modeling: a primer. Comm. Methods Meas. 2, 260–293. 10.1080/19312450802458935

[B30] Mottram A. J. Fleming M. J. (2009). Extraversion, impulsivity, and online group membership as predictors of problematic Internet use. CyberPsychol. Behav. 12, 319–321. 10.1089/cpb.2007.0170 19445635

[B31] Muthén L. Muthén B. (1998-2015). Mplus User's Guide, 6th Edn. Los Angeles, CA: Muthén & Muthén.

[B32] Nigg C. R. Mateo D. J. An J. (2017). Pokémon GO may increase physical activity and decrease sedentary behaviors. Am. J. Public Health 107, 37–38. 10.2105/AJPH.2016.303532 27854536 PMC5308176

[B33] Orosz G. Vallerand R. J. Bothe B. Tóth-Király I. Paskuj B. (2016). On the correlates of passion for screen-based behaviors: the case of impulsivity and the problematic and non-problematic facebook use and TV series watching. Pers. Indiv. Differ. 101, 167–176. 10.1016/j.paid.2016.05.368

[B34] Peterson C. M. Fischer S. (2012). A prospective study of the influence of the UPPS model of impulsivity on the co-occurrence of bulimic symptoms and non-suicidal self-injury. Eat Behav. 13, 335–341. 10.1016/j.eatbeh.2012.05.007 23121784

[B35] Przybylski A. K. Weinstein N. Ryan R. M. Rigby C. S. (2009). Having to versus wanting to play: background and consequences of harmonious versus obsessive engagement in video games. CyberPsychol. Behav. 12, 485–492. 10.1089/cpb.2009.0083 19772442

[B36] Rogers W. M. Schmitt N. (2004). Parameter recovery and model fit using multidimensional composites: a comparison of four empirical parceling algorithms. Multi. Behav. Res. 39, 379–412. 10.1207/S15327906MBR3903_1

[B37] Routledge C. Wildschut T. Sedikides C. Juhl J. (2013). Nostalgia as a resource for psychological health and well-being. Soc. Pers. Psychol. Comp. 7, 808–818. 10.1111/spc3.12070

[B38] Sedikides C. Wildschut T. Cheung W. Y. Routledge C. Hepper E. G. Arndt J.. (2016). Nostalgia fosters self-continuity: uncovering the mechanism (social connectedness) and consequence (eudaimonic well-being). Emotion 16, 524–539. 10.1037/emo0000136 26751632

[B39] statista.com (2017). Available online at: https://www.statista.com/statistics/665640/pokemon-go-global-android-apple-users/ (Accessed December 27, 2017).

[B40] Stoeber J. Harvey M. Ward J. A. Childs J. H. (2011). Passion, craving, and affect in online gaming: predicting how gamers feel when playing and when prevented from playing. Pers. Indiv. Differ. 51, 991–995. 10.1016/j.paid.2011.08.006

[B41] Tateno M. Skokauskas N. Kato T. A. Teo A. R. Guerrero A. P. (2016). New game software (Pokémon Go) may help youth with severe social withdrawal, hikikomori. Psychiatry Res. 246, 848–849. 10.1016/j.psychres.2016.10.038 27817905 PMC5573581

[B42] Tosun L. P. Lajunen T. (2009). Why do young adults develop a passion for internet activities? the associations among personality, revealing “true self” on the internet, and passion for the internet. CyberPsychol. Behav. 12, 401–406. 10.1089/cpb.2009.0006 19594378

[B43] Tóth-Király I. Bothe B. Rigó A. Orosz G. (2017). An illustration of the Exploratory Structural Equation Modeling (ESEM) framework on the passion scale. Front. Psychol. 8:1968. 10.3389/fpsyg.2017.01968 29163325 PMC5681952

[B44] Vallerand R. J. (2010). On passion for life activities: the dualistic model of passion. Adv. Exp. Soc. Psychol. 42, 97–193. 10.1016/S0065-2601(10)42003-1

[B45] Vallerand R. J. (2015). The Psychology of Passion: A Dualistic Model. New York, NY: Oxford University Press.

[B46] Vallerand R. J. Blanchard C. Mageau G. A. Koestner R. Ratelle C. Leonard M. (2003).Les passions de l'ame: on obsessive and harmonious passion. J. Pers. Soc. Psychol. 85, 756–767. 10.1037/0022-3514.85.4.756 14561128

[B47] Vallerand R. J. Rousseau F. L. Grouzet F. M. Dumais A. Grenier S. Blanchard C. M. (2006). Passion in sport: a look at determinants and affective experiences. J. Sport Exerc. Psychol. 28, 454–478. 10.1123/jsep.28.4.454

[B48] Wang C.-C. Chu Y.-S. (2007). Harmonious passion and obsessive passion in playing online games. Soc. Behav. Pers. 35, 997–1006. 10.2224/sbp.2007.35.7.997

[B49] Watanabe K. Kawakami N. Imamura K. Inoue A. Shimazu A. Yoshikawa T.. (2017). Pokémon GO and psychological distress, physical complaints, and work performance among adult workers: a retrospective cohort study. Sci. Rep. 7, 10758. 10.1038/s41598-017-11176-2 28883633 PMC5589944

[B50] Whiteside S. P. Lynam D. R. (2001). The five factor model and impulsivity: using a structural model of personality to understand impulsivity. Pers. Indiv. Differ. 30, 669–689. 10.1016/S0191-8869(00)00064-7

[B51] Yang C. C. Liu D. (2017). Motives matter: motives for playing Pokémon Go and implications for well-being. Cyberpsychol. Behav. Soc. Netw. 20, 52–57. 10.1089/cyber.2016.0562 28080150

[B52] Yee N. (2006). The demographics, motivations, and derived experiences of users of massively multi-user online graphical environments. Presence-Teleop. Virt. 15, 309–329. 10.1162/pres.15.3.309

[B53] Zsila Á. Bothe B. Demetrovics Z. Billieux J. Orosz G. (2017). Further exploration of the SUPPS-P Impulsive behavior scale's factor structure: evidence from a large hungarian sample. Curr. Psychol. 1–11. 10.1007/s12144-017-9773-7

